# Reviewed article: do Amaral VL, Spadotto GC, Gomes CB. Knowledge, attitudes and practices of primary care professionals about the food guide for children up to 2 years old: a cross-sectional study, Botucatu, São Paulo, Brazil, 2023. Epidemiol Serv Saúde. 2024;33:e20240111. doi: 10.1590/S2237-96222024v33e20240111.en

**DOI:** 10.1590/S2237-96222024v33e20240111.a

**Published:** 2024-12-13

**Authors:** Ariene Silva do Carmo

**Affiliations:** 1 Universidade Federal de Minas Gerais, Belo Horizonte, MG, Brazil

## Questionnaire

Does the manuscript contain new and significant information to justify publication? Yes

Does the Abstract (Summary) clearly and accurately describe the content of the article? Yes

Is the problem significant and concisely stated? Yes

Are the methods described comprehensively? Yes

Are the interpretations and conclusions justified by the results? Yes

Is adequate reference made to other work in the field? Yes

## Manuscript Structure

Length of article is: Adequate

Number of tables is: Adequate

Number of figures is: Adequate

**Please state any conflict(s) of interest that you have in relation to the review of this paper (state “none” if this is not applicable).**

None

**Interest:** Good

**Quality:** Excellent

**Originality:** Good

**Overall:** Good

**Recommendation:** Minor Revision

## Comments

O artigo é bastante relevante e está bem escrito. Entretanto, sugiro pequenos ajustes a serem realizados nos seguintes tópicos:

Padronizar ao longo do texto sobre o uso dos termos alimentação complementar (ou a forma abreviada: AM) e alimentação complementar (ou forma abreviada: AC)

## Introdução

- Linha 18: Sugiro substituir o trecho “doenças crônicas relacionadas à desnutrição ou à obesidade” por “todas as formas de má-nutrição e doenças crônicas relacionadas à alimentação inadequada”.
- Linha 25: Destacar a importância do aleitamento materno exclusivo até o sexto mês e continuado até 2 anos ou mais e a da introdução à alimentação complementar adequada e saudável a partir do sexto mês.
- Linha 41: Referenciar o trecho “Destaca-se que a maioria dos estudos, assim como este último, discorrem acerca do AM e AC e não especificamente sobre o Guia Alimentar”. Caso este trecho se refira aos dois estudos citados anteriormente e não há outro identificado pelos autores na literatura, sugiro reescrever o trecho para “Destaca-se que ambos os estudos discorrem acerca do AM e AC e não especificamente sobre o Guia Alimentar”.

## Métodos:

- Linha 9: Trazer mais informações sobre o instrumento que avaliou conhecimentos e atitudes referentes ao AM e AC. É um instrumento já validado em outro estudo? Ou foi criado pelos autores para a realização deste estudo? Se sim, houve validação deste instrumento?
- Em análises estatística: (1) adicionar que a análise descritiva contemplou o cálculo de frequências absolutas e relativas para as variáveis categóricas e de média e intervalo de confiança de 95% e valores mínimos e máximos para as variáveis quantitativas; (2) adicionar os pontos de cortes da categorização da variável número total de acertos.

## Resultados

- Linha 30: Retirar a palavra “maioria” deste trecho: “Quanto à formação, a maioria (24,3%) concluiu a graduação há menos de cinco anos...”, ficando da seguinte forma: “Quanto à formação, 24,3% concluiram a graduação há menos de cinco anos...”.
- No parágrafo que apresenta os dados das análises que usam a variável total de acertos, adicionar a seguinte informação em parêntese: (dados não apresentados)”.
- Tabelas: conferir os percentuais, pois algumas frequências relativas não somam exatamente 100% (a exemplo das variáveis raça/cor e escolaridade na Tabela 1

## Discussão

- Dentre as ações do Ministério da Saúde para qualificação profissional, citar também os cursos gratuitos sobre essa temática que estão disponíveis e com inscrições abertas na plataforma UNA-SUS e são voltados para profissionais da APS:

Amamenta e alimenta Brasil: recomendações baseadas no Guia Alimentar para Crianças Brasileiras Menores de 2 anos: https://www.unasus.gov.br/cursos/curso/46403

Reconhecendo a Norma Brasileira de Comercialização de Alimentos para Lactentes e Crianças de Primeira Infância, Bicos, Chupetas e Mamadeiras (NBCAL): formação para Profissionais da Rede de Atenção à Saúde: https://www.unasus.gov.br/cursos/curso/46786

O curso sobre guia da criança, por exemplo, está com inscrições abertas desde 2020.

- Também sugiro trazer na discussão alguns dados Botucatu (SP) que podem ser acessados nos relatórios públicos do Sisvan Web, tais como: cobertura de avaliação da situação alimentar e nutricional, do aleitamento materno (exclusivo e continuado), diversidade mínima e de consumo de ultraprocessados entre crianças de 6 a 23 meses. Esses dados podem apontar a necessidade de melhorar estes indicadores, reforçando a importância da qualificação dos profissionais de saúde no município.

